# Evidence for Significant Overlap between Common Risk Variants for Crohn's Disease and Ankylosing Spondylitis

**DOI:** 10.1371/journal.pone.0013795

**Published:** 2010-11-02

**Authors:** Debby Laukens, Michel Georges, Cécile Libioulle, Cynthia Sandor, Myriam Mni, Bert Vander Cruyssen, Harald Peeters, Dirk Elewaut, Martine De Vos

**Affiliations:** 1 Department of Gastroenterology, Ghent University, Ghent, Belgium; 2 Unit of Animal Genomics, GIGA-R and Faculty of Veterinary Medicine, University of Liège, Liège, Belgium; 3 Department of Rheumatology, Ghent University, Ghent, Belgium; HelmholtzZentrum München, Germany

## Abstract

**Background:**

A multicenter genome-wide association scan for Crohn's Disease (CD) has recently reported 40 CD susceptibility loci, including 29 novel ones (19 significant and 10 putative). To gain insight into the genetic overlap between CD and ankylosing spondylitis (AS), these markers were tested for association in AS patients.

**Principal Findings:**

Two previously established associations, namely with the *MHC* and *IL23R* loci, were confirmed. In addition, rs2872507, which maps to a locus associated with asthma and influences the expression of the *ORMDL3* gene in lymphoblastoid cells, showed a significant association with AS (p = 0.03). In gut biopsies of AS and CD patients, *ORMDL3* expression was not significantly different from controls and no correlation was found with the rs2872507 genotype (Spearman's rho: −0.067). The distribution of p-values for the remaining 36 SNPs was significantly skewed towards low p-values unless the top 5 ranked SNPs (*ORMDL3*, *NKX2–3, PTPN2*, *ICOSLG* and *MST1*) were excluded from the analysis.

**Conclusions:**

Association analysis using risk variants for CD led to the identification of a new risk variant associated with AS (*ORMDL3*), underscoring a role for ER stress in AS. In addition, two known and five potentially relevant associations were detected, contributing to common susceptibility of CD and AS.

## Introduction

Genome wide association studies (GWAS) are revealing increasing numbers of common risk variants for a growing list of pathologies. Common themes emerging from GWAS include (i) the polygenic architecture of most common diseases, including many risk variants with individually small effects, (ii) the absence of convincing epistatic interactions between common risk variants, and (iii) the fact that the common variants detected to date typically account for less than 20% of the genetic risk. Association mapping greatly benefits from meta-analyses, as pooling of medium sized cohorts considerably increases the power to detect the mainly small genetic effects [1].

An additional noteworthy outcome of recent GWAS are the connections that are being established between diseases initially considered unrelated, through the identification of shared risk variants. Examples include the association of *IL23R* variants with Crohn's disease (CD), psoriasis and ankylosing spondylitis, of *PTPN2* variants with CD and type 1 diabetes, and of *ORMDL3* variants with asthma and CD [2]–[7].

Crohn's disease and ankylosing spondylitis (AS) are idiopathic, chronic inflammatory disorders of, respectively, the intestinal tract and the spine and sacroiliac joints [8], [9]. Although very distinct and well defined entities, there is clinical and genetic evidence supporting some degree of overlap between the pathogenesis of the two entities. Crohn's disease is associated in up to 30% of the patients with articular pathology including sacroilliitis, spondylitis and/or peripheral arthritis [10]. Prior studies also demonstrated evidence for the presence of asymptomatic chronic intestinal inflammation in a subgroup of patients with spondylarthritis (SpA) associated with an increased risk for the development of CD [11]. *HLA-B27* has been known for a long time to be a major risk factor for AS [12], while the previously suspected influence of the MHC on CD susceptibility has recently been clearly confirmed [7]. As mentioned before, *IL23R* variants have been shown to be associated with both CD and AS [2], [4]. Risk variants for CD in *NOD2* have also been shown to increase the risk for chronic gut inflammation in SpA patients [13]. Finally, animal models including HLA-B27/human β2-microglobulin transgenic rats and TNFΔ^ARE^ mice, support links between articular and gut inflammation [14], [15].

Through a meta-analysis involving a total of 6,894 CD cases and 9,316 controls of Caucasian descent, we recently identified 19 novel CD risk loci in addition to 11 previously identified ones. Moreover, we presented strongly suggestive evidence for at least 10 more loci, for a total of 40 CD risk loci [7]. To further examine the potential overlap between the inherited susceptibility to CD and AS, we genotyped a cohort of 182 AS patients for SNP markers corresponding to 39 of these 40 CD risk loci described in Barrett et al. and evaluated their effect on AS outcome [7]. *NOD2* was not included in the analysis because we and others have shown that none of the three CD-associated SNPs are associated with AS [13], [16].

## Results

As expected, a highly significant association (p = 0.00004) was found with rs3763313, corresponding to the well established major effect of the *MHC* [12]. In addition, we observed a nominally significant association (p = 0.04) with rs11209026, which is in agreement with the previously described effect on AS of the *IL23R* locus (Supporting Table S1) [4].

Of the remaining 37 SNPs, rs2872507 showed a significant association with AS after Bonferroni correction for 37 new tests (p = 0.03), this being a strong candidate for a novel AS susceptibility locus. Because this SNP has been shown to influence the expression of the *ORMDL3* gene [7], we measured the transcript abundance of this gene in endoscopically healthy intestinal biopsies of patients with AS, CD and ulcerative colitis (UC) (patient characteristics Table 1). No difference was found in *ORMDL3* mRNA expression in the colon or ileum of CD, UC and AS patients as compared to healthy controls. In addition, no correlation was found between the expression of this gene and the rs2872507 genotype AA/AG/GG (Spearman's Rho: −147 for the total group, N = 82; 0.147 for ileal biopsies only, N = 32; 0.138 for colonic biopsies only, N = 50).

**Table 1:** Clinical features of the patient population recruited for intestinal ORMDL3 gene expression analysis.

	Colonic biopsies				Ileal biopsies			
	control	CD	UC	AS	control	CD	UC	AS
N	21	39	10	14	17	24	11	10
Gender (M/F)	9/12	18/21	7/3	8/6	8/9	12/12	4/7	4/6
Age, yrs (mean, range)	50 (22–69)	38 (11–73)	45 (25–61)	36 (16–51)	51 (27–69)	37 (11–66)	32 (7–51)	35 (16–44)
Age at diagnosis (A1/A2/A3)		3/28/8	0/4/6			3/15/6	1/8/2	
Disease location (L1/L2/L3/L4)		15/5/18/1				4/8/11/1		
Rs2872507 (AA/AG/GG/unknown)	3/6/6/6	9/11/1/18	0/3/0/7	0/7/4/3	2/3/5/7	3/9/1/11	0/1/0/10	0/5/3/2
Medication intake:								
no	21	30	7	2	17	19	10	1
5-aminosalicylates	0	9	3		0	5	1	
NSAID				12				9

A1:0–16 yrs; A2:16–40 yrs; A3: ≥40 yrs; disease location is defined as maximal extension of inflammation before first resection. L1: ileal involvement only, L2: colonic involvement only, L3: ileal and colonic involvement. NSAID: non-steroidal anti-inflammatory drugs.

**Figure 1:**
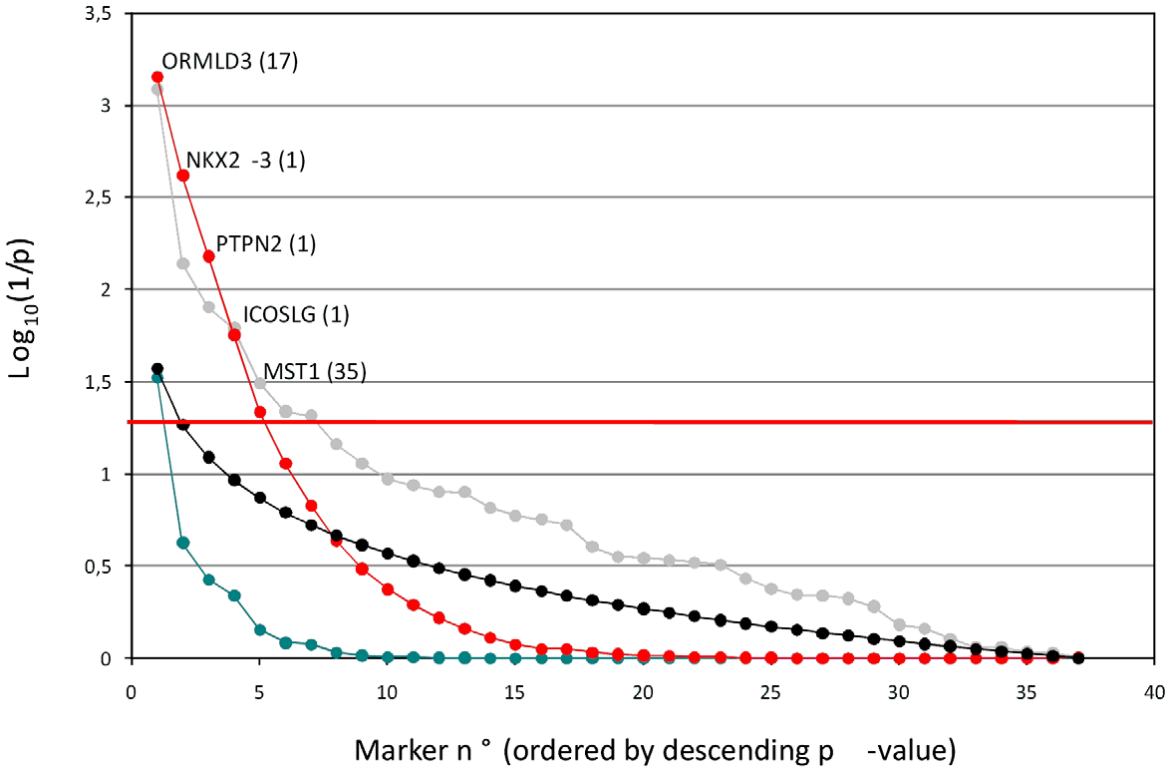
Association of 36 SNPs known to influence CD risk with AS. SNPs are ordered on the X-axis by increasing p-value. Y-axis: log_10_(1/p), corresponding to (i) nominal p-values (gray), (ii) Bonferroni corrected p-values (blue), (iii) expected distribution of p-values assuming that all SNPs are true null hypotheses (black), and (iv) the p-value of the distribution of individual p-values for the corresponding marker plus all the less significant ones (red). The horizontal line corresponds to a p-value of 0.05. The names of gene of interest in the vicinity of the associated SNPs as well as the number of genes in the confidence interval (defined according to [7]) are given for the five most interesting SNPs, exceeding the 0.05 significance threshold using the approach that extracts information from the p-value distribution.

None of the other 36 SNPs yielded significant p-values after Bonferroni correction, which was not really a surprise given the small size of the studied AS cohort. However, it is well established that one can gain additional statistical power by examining the distribution of p-values across a series of tests rather than considering each of them individually [17]. Thus, we verified whether the remaining 36 SNPs were showing an excess of low p-values when compared to a typical distribution of p-values expected for 36 true null hypotheses. The sum of log(1/*p_i_*) values obtained with the 36 SNPs was only reached for 7 out of 10,000 simulations, thus equating to a p-value of 0.0007, strongly suggesting the occurrence of true alternative hypotheses amongst the 36 remaining SNPs. The same approach was successively applied to the 35, 34, 33, 32, … SNPs with highest p-values, i.e. progressively dropping the SNP with lowest p-values and verifying whether the remaining distributions were still significantly skewed towards low values. The outcome of this analysis is shown in Fig. 1. It indicates that the top four SNPs, at least, are very likely to affect the risk of developing AS.

## Discussion

We herein first confirm two previously established associations with the risk to develop AS, respectively with the *MHC* and *IL23R* loci.

In addition, we report a novel association between AS and a SNP mapping at position 35,294,289 of human chromosome 17. Remarkably, rs2872507 maps to a locus shown first to be associated with asthma [6] and subsequently with CD [7]. Rs2872507 was shown to be associated with expression levels of the closely linked *ORMDL3* and *GSDML* genes in lymphoblastoid cell lines, which therefore stood out as prime candidate genes [6], [7]. However we could not detect differential expression of *ORMDL3* in whole gut biopsies of patients with CD or AS. Although this polymorphism has been shown to influence the expression of *ORMDL3* in lymphoblastoid cells, it is likely that such influences might play only a marginal role in complex tissue such as gut biopsies. Alternatively, SNPs in LD with rs2872507 might have more profound control on the expression of ORMDL3.

Using a survey of SNPs surrounding the *ORMDL3* gene genotyped in the Welcome Trust case consortium dataset, no association was found with AS (personal communication). The population used in this study might contain a bias towards patients with subclinical gut alterations, since the patients were sampled as the result of a collaboration between the departments of rheumatology and gastroenterology. *HLA-B27*, a well-known risk factor associated with AS, has the tendency to misfold during class I complex formation in the endoplasmic reticulum (ER). As such, *ORMDL3* represents an interesting candidate gene for AS, as this protein resides in the ER and overexpression of this gene facilitates the activation of the unfolded protein response [18].

By applying a method that seeks to extract information from the distribution of p-values rather than individual ones, we provide evidence for an addition of four novel AS risk loci. The three first of these define an LD-based confidence interval encompassing one gene each: *NKX2-3, PTPN2* and *ICOSLG* [7]. As mentioned before, *PTPN2* is particularly interesting as it has been implicated before in the pathogenesis of CD [7] and type I diabetes [19]. The sign of the association is apparently the same for the three diseases, i.e. the same allele increases risk for the three pathologies. *ICOSLG* is also very interesting because of its known involvement in the regulation of immune response [20], [21]. The fourth SNP defines a confidence interval encompassing 35 genes, including *MST1* which has recently been implicated in the pathogenesis of CD [22].

One could argue that the observed skewed distribution of p-values reflects population stratification rather than genuine associations. While we cannot formally refute this possibility on the basis of the available data, we consider this to be an unlikely hypothesis. Indeed, the controls originated from the same geographical region as the cases, namely Belgium, and were subjected to the same ethnicity criteria. Moreover, the exact same control cohort has been successfully used as confirmation cohort in an association study for CD based on Belgian cases [7].

In conclusion, we herein provide evidence for an important overlap between the determinants of inherited predisposition to CD and AS. Prior to this study the *MHC* and *IL23R* loci were known to be implicated in the susceptibility to both diseases. We have studied an additional 37 recently defined CD risk loci. Given the limited size of the studied AS cohort, the significance threshold associated with a Type-I error of 5% (accounting for the realization of 37 independent tests), was only exceeded for one (*ORMLD3*) of the 37 SNPs not previously known to affect AS. However, the distribution of p-values for the remaining 36 SNPs was significantly skewed towards low p-values unless the top 5 SNPs were removed from the analysis, hence supporting at least five novel associations with AS.

## Materials and Methods

### Ethics Statement

This study was approved by the ethics committee of the University Hospital Ghent (nos. 2000/242 and 2004/242) and each participant obtained a written informed consent form. This form was signed by the participants and approved by the ethics committee.

### Patients

All included patients fulfilled the modified New York criteria for definite AS [23], were of self-reported white ancestry, born between 1930 and 1986. Patients were followed at the Rheumatology Department of the University Hospital in Ghent. The male to female ratio was 2.4. Patients with abdominal inflammatory symptoms were not included in this analysis.

### Genotyping

Genotyping was performed using the Illumina Golden Gate assay previously used on the Belgian-French cohort in the CD meta-analysis [7]. The genotyping success rate for the AS patients that were retained for analysis was ≥97% and all markers were in Hardy-Weinberg equilibrium in the control population [7].

### Quantitative real-time PCR

For *ORMDL3* gene expression analysis, endoscopically healthy biopsies were retrieved during colonoscopy. Total RNA was extracted from the biopsies using the RNeasy Mini Kit (Qiagen Benelux, Venlo, The Netherlands). The quality of each sample was determined using automated gelelectrophoresis (Experion Systems, Maynard, MA, USA, RQI range 7.6–10). Twenty ng of total RNA was converted to cDNA and amplified using the WT-Ovation RNA amplification system (Nugen Technologies, Bemmel, The Netherlands). Ten ng of amplified cDNA was used in SYBRGreen real-time quantitative PCR using automated pipetting (Caliper ALH3000, Caliper Life Sciences, Teralfene, Belgium). Cycling conditions were 95°C for 10 minutes and 44 cycles of 95°C for 10 seconds and 60°C for 30 seconds (Bio-Rad CFX384, Bio-Rad Laboratories, Nazareth, Belgium). Melting curve analysis confirmed primers specificities. The amplification efficiencies of the primer pairs were calculated using a standard curve of reference genomic DNA. Amplification efficiency was determined using the formula 10^−1/slope^. *ORMDL3* expression was normalized with the geometric mean value of three reference genes. Primer sequences for *ORMDL3* detection were GTAAAAGGCATGTGCTGCAA; CCCAACCCCACTACAAGCTA (E = 105% R^2^ = 0.999), for GAPDH TGCACCACCAACTGCTTAGC; GGCATGGACTGTGGTCATGAG (E = 110%; R^2^ = 0.990), for HPRT TGACACTGGCAAAACAATGCA; GGTCCTTTTCACCAGCAAGCT (E = 111%; R^2^ = 0.998) and for SDHA TGGGAACAAGAGGGCATCTG; CCACCACTGCATCAAATTCATG (E = 105; R^2^ = 0.994).

### Statistics

Marker allele frequencies were compared between AS cases and previously described Belgian replication controls, using a one-sided Fisher's exact test imposing an allelic effect with the same sign as observed for CD [7]. For the 36 SNPs that did not exceed Bonferroni-adjusted significance thresholds, we compared the distribution of p-values with that expected for 36 true null hypotheses. This was achieved by comparing 
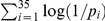
 obtained with the real data, with the distribution of 10,000 
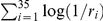
, where *r_i_* are random numbers drawn between 0 and 1. Gene expression differences between groups were evaluated by the Kruskal-Wallis statistic with Dunn's multiple comparison test.

## Supporting Information

Table S1 (0.22 MB DOC)
