# Supplementary material for: Evidence for Significant Overlap between Common Risk Variants for Crohn's Disease and Ankylosing Spondylitis
Source: PLoS One. 2010 Nov 2;5(11):e13795. doi: 10.1371/journal.pone.0013795 (PMC2970560; doi:10.1371/journal.pone.0013795)
Supplement: Table S1 — (0.22 MB DOC) [file pone.0013795.s001.doc]

**Supporting information:** Association between 39 SNPs known to be associated with CD and AS

|  | | CTRL | | | | | CD | | | | | AS | | | | | Haplotype RR | | Association p-value | | | |  |
| --- | --- | --- | --- | --- | --- | --- | --- | --- | --- | --- | --- | --- | --- | --- | --- | --- | --- | --- | --- | --- | --- | --- | --- |
| SNP | CHR | GT1 | GT2 | GT3 | f(A) | n | GT1 | GT2 | GT3 | f(A) | n | GT1 | GT2 | GT3 | f(A) | n | CD | AS | CD | AS | AS | AS | Genes of interest |
|  |  |  |  |  |  |  |  |  |  |  |  |  |  |  |  |  |  |  |  | (N) | (BFC) | (DIS) |  |
| rs3763313 | 6 | 0.025 | 0.32 | 0.65 | 0.19 | 693 | 0.046 | 0.36 | 0.59 | 0.23 | 1071 | 0.1 | 0.36 | 0.53 | 0.29 | 173 | 1.283 | 1.752 | 0.00404 | 0.00004 |  |  | BTNL2 |
| rs2872507 | 17 | 0.336 | 0.47 | 0.19 | 0.57 | 693 | 0.249 | 0.5 | 0.25 | 0.5 | 1071 | 0.25 | 0.45 | 0.3 | 0.48 | 173 | 1.342 | 1.467 | 0.00002 | 0.00083 | 0.03012 | 0.00070 | ORMDL3 |
| rs11190140 | 10 | 0.278 | 0.47 | 0.25 | 0.51 | 676 | 0.226 | 0.5 | 0.27 | 0.48 | 1051 | 0.22 | 0.43 | 0.35 | 0.44 | 167 | 1.15 | 1.354 | 0.04689 | 0.00725 | 0.23604 | 0.00240 | NKX2-3 |
| rs2542151 | 18 | 0.033 | 0.23 | 0.74 | 0.15 | 692 | 0.031 | 0.3 | 0.67 | 0.18 | 1071 | 0.05 | 0.29 | 0.66 | 0.2 | 173 | 1.286 | 1.431 | 0.00833 | 0.01255 | 0.37332 | 0.00660 | PTPN2 |
| rs762421 | 21 | 0.157 | 0.49 | 0.36 | 0.4 | 693 | 0.165 | 0.5 | 0.34 | 0.41 | 1071 | 0.21 | 0.5 | 0.28 | 0.47 | 173 | 1.06 | 1.303 | 0.42007 | 0.01630 | 0.45565 | 0.01760 | ICOSLG |
| rs9858542 | 3 | 0.567 | 0.37 | 0.07 | 0.75 | 700 | 0.506 | 0.38 | 0.11 | 0.7 | 1087 | 0.48 | 0.45 | 0.07 | 0.7 | 174 | 1.313 | 1.284 | 0.00046 | 0.03244 | 0.70483 | 0.04620 | MST1 |
| rs11209026 | 1 | 0.872 | 0.12 | 0 | 0.93 | 693 | 0.947 | 0.05 | 0 | 0.97 | 1071 | 0.92 | 0.07 | 0.01 | 0.96 | 173 | 2.51 | 1.686 | 0.00000 | 0.03952 |  |  | IL23R |
| rs1456893 | 7 | 0.102 | 0.44 | 0.46 | 0.32 | 693 | 0.079 | 0.42 | 0.5 | 0.29 | 1071 | 0.08 | 0.39 | 0.53 | 0.27 | 173 | 1.167 | 1.262 | 0.03923 | 0.04601 | 0.82500 | 0.08720 |  |
| rs4807569 | 19 | 0.055 | 0.35 | 0.6 | 0.23 | 703 | 0.045 | 0.32 | 0.63 | 0.21 | 1055 | 0.05 | 0.28 | 0.67 | 0.19 | 175 | 1.139 | 1.297 | 0.12116 | 0.04847 | 0.84089 | 0.14890 |  |
| rs4613763 | 5 | 0.004 | 0.22 | 0.78 | 0.11 | 693 | 0.029 | 0.3 | 0.67 | 0.18 | 1071 | 0.02 | 0.24 | 0.73 | 0.14 | 173 | 1.687 | 1.313 | 0.00000 | 0.06931 | 0.92989 | 0.22940 | PTGER4 |
| rs7849191 | 9 | 0.359 | 0.48 | 0.16 | 0.6 | 693 | 0.393 | 0.47 | 0.14 | 0.63 | 1070 | 0.41 | 0.46 | 0.13 | 0.64 | 173 | 1.116 | 1.189 | 0.12729 | 0.08784 | 0.96668 | 0.32730 | JAK2 |
| rs7927894 | 11 | 0.343 | 0.52 | 0.14 | 0.6 | 702 | 0.333 | 0.49 | 0.17 | 0.58 | 1076 | 0.33 | 0.47 | 0.2 | 0.56 | 165 | 1.097 | 1.17 | 0.18559 | 0.10647 | 0.98448 | 0.42240 | C11Orf30 |
| rs11175593 | 12 | 0.958 | 0.04 | 0 | 0.98 | 693 | 0.947 | 0.05 | 0 | 0.97 | 1066 | 0.94 | 0.06 | 0 | 0.97 | 173 | 1.262 | 1.536 | 0.36883 | 0.11565 | 0.98940 | 0.51410 | LRRK2,MUC19 |
| rs8098673 | 18 | 0.102 | 0.47 | 0.43 | 0.34 | 693 | 0.105 | 0.45 | 0.44 | 0.33 | 1070 | 0.09 | 0.42 | 0.49 | 0.3 | 173 | 1.022 | 1.163 | 0.76992 | 0.12539 | 0.99297 | 0.60500 |  |
| rs2301436 | 6 | 0.316 | 0.47 | 0.21 | 0.55 | 693 | 0.225 | 0.48 | 0.29 | 0.47 | 1071 | 0.28 | 0.47 | 0.25 | 0.52 | 173 | 1.407 | 1.149 | 0.00000 | 0.12606 | 0.99316 | 0.69250 | CCR6 |
| rs6908425 | 6 | 0.605 | 0.35 | 0.04 | 0.78 | 693 | 0.653 | 0.31 | 0.03 | 0.81 | 1071 | 0.65 | 0.32 | 0.03 | 0.81 | 173 | 1.203 | 1.175 | 0.03176 | 0.15300 | 0.99785 | 0.77630 | CDKAL1 |
| rs17309827 | 6 | 0.128 | 0.44 | 0.43 | 0.35 | 690 | 0.132 | 0.41 | 0.45 | 0.34 | 1056 | 0.11 | 0.4 | 0.48 | 0.31 | 99 | 1.049 | 1.177 | 0.53535 | 0.16899 | 0.99894 | 0.84190 | SLC22A23 |
| rs7758080 | 6 | 0.062 | 0.45 | 0.49 | 0.29 | 693 | 0.088 | 0.46 | 0.45 | 0.32 | 1070 | 0.08 | 0.47 | 0.45 | 0.31 | 173 | 1.164 | 1.134 | 0.04720 | 0.17718 | 0.99927 | 0.89690 |  |
| rs1551398 | 8 | 0.138 | 0.45 | 0.41 | 0.37 | 658 | 0.088 | 0.46 | 0.45 | 0.32 | 1070 | 0.08 | 0.47 | 0.45 | 0.31 | 173 | 1.237 | 1.269 | 0.91328 | 0.18912 | 0.99957 | 0.89630 |  |
| rs10995271 | 10 | 0.375 | 0.46 | 0.16 | 0.61 | 693 | 0.306 | 0.49 | 0.2 | 0.55 | 1071 | 0.38 | 0.42 | 0.2 | 0.59 | 173 | 1.252 | 1.09 | 0.00134 | 0.24970 | 0.99998 | 0.93370 | ZNF365 |
| rs4263839 | 9 | 0.471 | 0.41 | 0.12 | 0.68 | 692 | 0.306 | 0.49 | 0.2 | 0.55 | 1071 | 0.49 | 0.4 | 0.1 | 0.69 | 173 | 1.69 | 0.923 | 0.00145 | 0.28121 | 1.00000 | 0.95480 | TNFSF15 |
| rs3764147 | 13 | 0.043 | 0.38 | 0.58 | 0.23 | 693 | 0.069 | 0.41 | 0.52 | 0.27 | 1071 | 0.05 | 0.39 | 0.56 | 0.25 | 173 | 1.258 | 1.08 | 0.00403 | 0.28562 | 1.00000 | 0.96780 |  |
| rs2188962 | 5 | 0.318 | 0.46 | 0.22 | 0.55 | 663 | 0.306 | 0.49 | 0.2 | 0.55 | 1071 | 0.3 | 0.46 | 0.24 | 0.53 | 178 | 1.009 | 0.934 | 0.10067 | 0.29484 | 1.00000 | 0.97790 |  |
| rs2274910 | 1 | 0.478 | 0.42 | 0.1 | 0.69 | 703 | 0.515 | 0.41 | 0.08 | 0.72 | 1056 | 0.51 | 0.38 | 0.11 | 0.7 | 177 | 1.163 | 1.077 | 0.04524 | 0.30344 | 1.00000 | 0.98590 | ITLN1 |
| rs13003464 | 2 | 0.161 | 0.48 | 0.36 | 0.4 | 647 | 0.178 | 0.52 | 0.3 | 0.44 | 1068 | 0.18 | 0.47 | 0.35 | 0.41 | 176 | 1.183 | 1.069 | 0.02051 | 0.31195 | 1.00000 | 0.99170 | PUS10 |
| rs10045431 | 5 | 0.502 | 0.41 | 0.08 | 0.71 | 693 | 0.525 | 0.41 | 0.07 | 0.73 | 1071 | 0.52 | 0.4 | 0.08 | 0.72 | 173 | 1.094 | 1.052 | 0.24940 | 0.37019 | 1.00000 | 0.99460 | IL12B |
| rs11584383 | 1 | 0.078 | 0.39 | 0.53 | 0.27 | 703 | 0.063 | 0.37 | 0.57 | 0.25 | 1053 | 0.07 | 0.4 | 0.53 | 0.27 | 169 | 1.155 | 1.035 | 0.06972 | 0.41914 | 1.00000 | 0.99670 |  |
| rs12529198 | 6 | 0.001 | 0.14 | 0.86 | 0.07 | 693 | 0.003 | 0.12 | 0.87 | 0.06 | 1071 | 0.01 | 0.12 | 0.87 | 0.07 | 173 | 1.141 | 1.055 | 0.33742 | 0.45386 | 1.00000 | 0.99780 | LYRM4 |
| rs2476601 | 1 | 0.82 | 0.17 | 0.01 | 0.9 | 693 | 0.859 | 0.13 | 0.01 | 0.93 | 1071 | 0.83 | 0.15 | 0.02 | 0.91 | 172 | 1.324 | 1.035 | 0.02425 | 0.45931 | 1.00000 | 0.99910 | PTPN22 |
| rs3828309 | 2 | 0.289 | 0.5 | 0.21 | 0.54 | 705 | 0.374 | 0.47 | 0.16 | 0.61 | 1045 | 0.31 | 0.47 | 0.23 | 0.54 | 172 | 1.338 | 1.013 | 0.00003 | 0.47596 | 1.00000 | 0.99900 | ATG16L1 |
| rs744166 | 17 | 0.189 | 0.48 | 0.33 | 0.43 | 693 | 0.151 | 0.45 | 0.39 | 0.38 | 1071 | 0.18 | 0.5 | 0.32 | 0.43 | 173 | 1.233 | 0.992 | 0.00309 | 0.52418 | 1.00000 | 0.99970 | STAT3 |
| rs991804 | 17 | 0.556 | 0.36 | 0.08 | 0.74 | 693 | 0.564 | 0.38 | 0.06 | 0.75 | 1071 | 0.53 | 0.4 | 0.08 | 0.73 | 173 | 1.083 | 0.941 | 0.32189 | 0.65854 | 1.00000 | 1.00000 | CCL2,CCL7 |
| rs917997 | 2 | 0.597 | 0.35 | 0.05 | 0.77 | 693 | 0.569 | 0.38 | 0.06 | 0.76 | 1071 | 0.6 | 0.37 | 0.03 | 0.79 | 173 | 1.087 | 0.917 | 0.31177 | 0.69256 | 1.00000 | 0.99990 | IL18RAP |
| rs7746082 | 6 | 0.084 | 0.42 | 0.5 | 0.29 | 693 | 0.101 | 0.45 | 0.45 | 0.32 | 1071 | 0.03 | 0.47 | 0.5 | 0.27 | 173 | 1.158 | 0.89 | 0.05287 | 0.78716 | 1.00000 | 0.99990 |  |
| rs1736135 | 21 | 0.183 | 0.51 | 0.31 | 0.44 | 693 | 0.181 | 0.47 | 0.35 | 0.42 | 1071 | 0.18 | 0.58 | 0.24 | 0.47 | 173 | 1.097 | 0.867 | 0.18532 | 0.87459 | 1.00000 | 1.00000 |  |
| rs11747270 | 5 | 0.009 | 0.17 | 0.82 | 0.09 | 693 | 0.022 | 0.21 | 0.77 | 0.13 | 1071 | 0 | 0.14 | 0.86 | 0.07 | 173 | 1.405 | 0.759 | 0.00272 | 0.87682 | 1.00000 | 0.99990 | IRGM |
| rs17582416 | 10 | 0.11 | 0.46 | 0.43 | 0.34 | 692 | 0.159 | 0.44 | 0.4 | 0.38 | 1070 | 0.08 | 0.44 | 0.49 | 0.29 | 173 | 1.21 | 0.821 | 0.00887 | 0.92936 | 1.00000 | 0.99960 |  |
| rs9286879 | 1 | 0.052 | 0.36 | 0.58 | 0.23 | 693 | 0.061 | 0.41 | 0.52 | 0.27 | 1070 | 0.03 | 0.32 | 0.65 | 0.19 | 173 | 1.201 | 0.787 | 0.02179 | 0.94287 | 1.00000 | 0.99710 |  |
| rs780094 | 2 | 0.345 | 0.48 | 0.17 | 0.59 | 693 | 0.332 | 0.48 | 0.18 | 0.57 | 1071 | 0.43 | 0.44 | 0.13 | 0.65 | 173 | 1.054 | 0.753 | 0.46357 | 0.98643 | 1.00000 | 0.98690 | GCKR |

“Genotypes” defines the three possible genotypes for each SNP; “CTRL”, “CD”, “AS” show the frequency of the three corresponding genotypes in controls, CD and AS patients respectively, with indication of the successfully genotyped sample size (“n”). “Association” provides the outcome of the association study using Fisher’s exact test. “CD”: association with CD; “AS(N)”: nominal p-values for the association with AS; “SA(BFC)”: Bonferroni corrected (37 tests – cfr. Text) p-values for the association with AS; “AS(DIS)”: p-value of the distribution of p-values for the corresponding SNP plus all less significant ones.
